# Improvement of a Predictive Model of Castration-Resistant Prostate Cancer: Functional Genetic Variants in TGFβ1 Signaling Pathway Modulation

**DOI:** 10.1371/journal.pone.0072419

**Published:** 2013-08-09

**Authors:** Ana L. Teixeira, Mónica Gomes, Augusto Nogueira, Andreia S. Azevedo, Joana Assis, Francisca Dias, Juliana I. Santos, Francisco Lobo, António Morais, Joaquina Maurício, Rui Medeiros

**Affiliations:** 1 Molecular Oncology Group, Portuguese Institute of Oncology - Porto, Porto, Portugal; 2 Abel Salazar Institute for the Biomedical Sciences - University of Porto, Porto, Portugal; 3 Urology Department, Portuguese Institute of Oncology - Porto, Porto, Portugal; 4 Oncology Department, Portuguese Institute of Oncology - Porto, Porto, Portugal; 5 . Faculty of Health Sciences of Fernando Pessoa University, Porto, Portugal; 6 . Research Department, Portuguese League Against Cancer (NRNorte), Porto, Portugal; Queensland University of Technology, Australia

## Abstract

Prostate cancer (PC) is the most frequently diagnosed cancer in men. The acquisition of castration-resistant (CR) phenotype is associated with the activation of signaling pathways mediated by growth factors. The TGFβ1 and its receptors have an important role in tumor progression, being the pro-apoptotic function modulated by the expression of *TGFBR2*. A single nucleotide polymorphism -875 G > A in *TGFBR2* gene has been described, which may influence the expression levels of the receptor. Our purpose was to investigate the potential role of *TGFBR2-875G>A* in PC risk and in the response to androgen deprivation therapy (ADT). *TGFBR2-875G>A* polymorphism was studied by allelic discrimination using real-time polymerase chain reaction (PCR) in 891 patients with PC and 874 controls. A follow-up study was undertaken to evaluate response to ADT. The *TGFBR2* and *SMAD7* mRNA expression were analyzed by a quantitative real-time PCR. We found that *TGFBR2-875GG* homozygous patients present lower expression levels of *TGFBR2* mRNA (AA/AG: 2^-ΔΔCT^ =1.5, *P*=0.016). GG genotype was also associated with higher Gleason grade (OR=1.51, *P*=0.019) and increased risk of an early relapse after ADT (HR=1.47, *P*=0.024). The concordance (c) index analysis showed that the definition of profiles that contains information regarding tumor characteristics associated with genetic information present an increased capacity to predict the risk for CR development (c-index model 1: 0.683 *vs* model 2: 0.736 *vs* model 3: 0.746 *vs* model 4: 0.759). The *TGFBR2-875G>A* contribution to an early relapse in ADT patients, due to changes in mRNA expression, supports the involvement of TGFβ1 pathway in CRPC. Furthermore, according to our results, we hypothesize the potential benefits of the association of genetic information in predictive models of CR development.

## Introduction

Prostate cancer (PC) is an important public health problem that affects the male population. After lung cancer, PC is the most frequently diagnosed cancer in men, the fifth cause of death by cancer worldwide, and nearly three-quarters of the registered cases occur in developed countries [1]. The causes of PC remain poorly understood and many gene products show deregulated functions during cancer progression. At diagnosis, patients with early stages of disease are frequently submitted to prostatectomy, external radiation and/or brachytherapy, which removes or destroys tumoral cells that are confined within the prostate [2]. However, despite recent advances in the early detection of localized PC tumors, there is little effective therapy for patients with locally advanced and/or metastatic disease. Patients diagnosed in advanced stages are currently submitted to androgen deprivation therapy (ADT), due to the androgen dependency of prostate cells for continued growth and survival. However, it was found that in most patients the effects of this therapy typically last 18 to 24 months, after which the patients develop resistance to hormonal therapy and develop castration-resistant prostate cancer (CRPC) [3]. Unfortunately, the CRPC treatment is limited, ineffective and the molecular mechanisms of its phenotype progression are not well understood. The CRPC is an invariably lethal condition, which frequently metastasize and is associated with a significant morbility and mortality [4].

Prostate cells require androgens in the cellular microenvironment to proliferate and differentiate. Nevertheless, PC progression and the acquisition of castration-resistant (CR) phenotypes have been associated with the activation of other signaling pathways mediated by growth factors that modulate the balance between the cell growth rate and apoptosis.

The TGFβ1 and its receptors are key components of the TGFβ signaling pathway, which has an important role in carcinogenesis and tumor progression. The signal transduction initiates with the TGFβ1 activation, then TGFβ1 binds to the type II receptor (TGFβRII), which then phosphorylates the type I receptor (TGFβRI), and activates its kinase. Phosphorylated TGFβRI, in its turn phosphorylates downstream elements of the signaling pathway. However the inhibitory SMAD7 has the capacity to bind to TGFβRI and effectively attenuate pathway activation [5]. *In vitro* studies have shown that in PC cells, the TGFβ1 signaling pathway has some defects and the restoration of this pathway can suppress tumor growth by inhibiting cell proliferation [6,7]. Reduced *TGFBR2* expression levels are correlated with a shorter survival rate of colon cancer patients, as does the reduced expression of the co-receptor betaglycan in breast and PC patients [8,9]. High expression levels of *TGFBR2* can mediate the pro-apoptotic function of the TGFβ1 signaling pathway and its loss promotes invasion and malignant transformation [10,11].

Alterations in *TGFBR2* levels, with impact in the TGF β1 signaling pathway, might be involved in PC development/progression. A G > A transition in the -875 promoter position of the *TGFBR2* gene was reported and it may enhance transcription activity in normal epithelial cells and might increase the expression of *TGFBR2* gene [12]. Genetic variants, which modulate *TGFBR2* expression, may have impact in PC development and prognosis. The present study is the first to evaluate the relevance of the *TGFBR2-875G>A* (rs3087465) functional polymorphism in CRPC patients.

## Material and Methods

### Ethics statement

The study was conducted according to the principles of the Helsinki Declaration. The study was approved by the local ethics committee at the Portuguese Institute of Oncology of Porto (Portugal). All individuals signed a written informed consent to participate in the study.

### Study Population

This case–control study was performed in 891 patients, with a mean age of 66.2 (7.7), with histopathologically diagnosed PC at the Portuguese Institute of Oncology of Porto (Portugal). Patients’ disease stage distribution at the time of diagnosis was as follows: 53.2% presented localized disease (T1-T2b), 32.7% had locally advanced disease (T3-T4), and 14.2% had metastatic disease (N^+^ and/or M^+^). Cumulatively, a follow-up study (n=428) was undertaken to evaluate response to ADT. The types of hormonal treatment were as follows: anti-androgens plus luteinizing hormonereleasing hormone agonists (aLHRH) combination therapy (64.2%), aLHRH alone (5.4%), and anti-androgens alone (30.4%). Hormone resistance was evaluated through PSA recurrence, which was defined as two consecutive increasing PSA values more than 1.0 ng ml^-^1 and differing by more than 0.2 ng ml^-1^ [13]. The median follow-up time was 51 months.

Men older than 40 years of age, without known history of cancer were recruited from the Portuguese Institute of Oncology of Porto Centre Blood Donor’s Bank and included in the control group (n=874), with a mean age of 44.2 (13.7). Peripheral venous blood samples (8 mL) were collected from each subject enrolled in the study.

### TGFBR2 -875G>A polymorphism (rs3087465) genotyping

After DNA extraction using the QIAamp DNA Mini kit (Qiagen^®^) according to the manufacturer’s protocol, the *TGFBR2-875G>A* polymorphism (rs3087465) was analyzed by allelic discrimination using 7300 real-time PCR System (Applied Biosystems^®^). The reaction was based on a 5' nuclease PCR assay, using a TaqMan assay, which includes two allele-specific TaqMan ^®^MGB probes (Applied Biosystems^®^) containing distinct fluorescent dyes and a PCR primer pair to detect the specific *TGFBR2-875 G*>*A* single nucleotide polymorphism (SNP). Real-time PCR was carried out using a 6 µL reaction mixture, containing 1x Master Mix (Applied Biosystems^®^), with 1x probes (TaqMan ^®^assay C__27491740_10, Applied Biosystems^®^) and 20 ng of the DNA sample. Thermal conditions were 95^°^ C during 10 minutes for DNA polymerase activation, followed by 45 PCR cycles at 92^°^ C for 15 seconds and 60^°^ C for 1 minute. Quality control procedures implemented for genotype analyses included double sampling in 10% of the samples to assess reliability and the use of negative controls to step-away false positives. Two authors obtained the results independently, and the ambiguous results were reanalyzed.

### Gene expression profiling

The *TGFBR2* and *SMAD7* mRNA expression levels were analyzed by a quantitative real-time PCR. After *TGFBR2-875G>A* genotyping, 33 patients were randomly chosen among the patients submitted to ADT, and total cellular RNA was isolated. Initially, the RNA was isolated by TriPure reagent (Roche ^®^Applied Science), and after separation of the RNA fraction, the samples were purified using the commercial kit GeneJET™ RNA Purification Kit (Fermentas^®^). RNA samples were then used as a templates for cDNA synthesis, using a High Capacity RNA-to-cDNA Kit (Applied Biosystems^®^). Finally, reactions were carried out on a StepOne ^TM^One qPCR machine, containing 1x Master Mix (Applied Biosystems^®^), with 1x probes (TaqMan ^®^Gene Expression assays, Hs00234253_m1 and Hs00998193_m1, Applied Biosystems^®^), cDNA sample, and human GUSB (Beta Glucuronidase) endogenous control (Applied Biosystems^®^) was used to normalize the results, regarding the two biomarkers, since it presents a constant expression level. The data analysis was carried out using the StepOne Software v 2.2 (Applied Biosystems^®^) with the same baseline and threshold set for each plate, in order to generate threshold cycle (*Ct*) values for all the genes in each sample. The mRNA quantification was performed in triplicate and the results were confirmed by two independent investigators.

### Statistical analysis

Data analysis was performed by the computer software IBM®SPSS ^®^Statistics for Windows (Version 20.0). The odds ratio (OR) and its 95% confidence interval (95% CI) were calculated as a measurement of the association between *TGFBR2-875G>A* genotypes and the PC risk. The Hardy–Weinberg equilibrium was tested by a Pearson chi-square analysis to compare the observed versus the expected genotype frequencies. A Cox proportional hazard model was used to analyze the time to CT (determined by the interval of time since the beginning of ADT until CR or the last clinical visit), considering as covariates, tumor stage (clinically localized *versus* locally advanced *versus* distant metastases, European Association of Urology (EAU) Guidelines [14]), Gleason Score (≥8), and PSA levels at diagnosis. Cox regression models were used to adjust for potential confounder. The 2^-ΔΔCt^ method, along with Student’s t-test, was used in order to evaluate any statistical differences in the normalized expression of the *TGFBR2* and *SMAD7* mRNA here explored, between the different *TGFBR2-875 G*>*A* genotypes. The concordance (c) index was used to compare the predictive ability of the association of well-known prognostic variables with the *TGFBR2-875 GG* genotype, with c>0.5 being considered with a good prediction ability [15].

## Results

The *TGFBR2-875G>A* polymorphism genotype distribution in patients and controls is described in Table 1. The frequencies for AA/AG and GG genotypes were, respectively, 0.37 and 0.63 for PC patients and 0.38 and 0.62 in the control group. Observed *versus* expected genotype frequencies were calculated, and no deviation from Hardy–Weinberg equilibrium was observed (PC group, *P*=0.877, control group, *P*=0.328). We observed no statistical significant association between the *TGFBR2-875G>A* polymorphism and PC risk (OR=1.05; 95% IC: 0.87-1.28; *P*=0.645) (Table 1).

**Table 1:** *TGFBR2-875G>A* polymorphism-related odds ratio for PC and genotype frequencies in patients and controls.

	**PC group**	**Control group**	**OR**	**95% CI**	***P***
***TGFBR2-875G>A***					
AA	34 (0.04)	46 (0.05)			
AG	295 (0.33)	287 (0.33)	1.39	0.87-2.24	0.210
GG	562 (0.63)	541 (0.62)	1.41	0.89-2.34	0.179
AA/AG	329 (0.37)	333 (0.38)			
GG	562 (0.63)	541 (0.62)	1.05	0.87-1.28	0.645

PC, Prostate cancer; OR, odds ratio; 95% CI, 95% confidence interval

Within the group of patients, the *TGFBR2-875GG* genotype was associated with higher Gleason grades (≥8) (OR=1.51, 95%CI: 1.07-2.16, *P*=0.019). Additionally, although the association was not statistically significant, there was a trend to an overrepresentation of the GG genotype in the CR group compared with the individuals without CR (OR= 1.41; 95%CI: 0.95-2.08; *P* = 0.084) (Table 2).

**Table 2:** Aggressive phenotype disease according *TGFBR2-875G>A* polymorphism.

	**Gleason Score**		**OR**	**95% CI**	***P***
***TGFBR2-875G>A***	**<8**	**≥8**			
**AA/AG**	274 (0.39)	55 (0.30)			
**GG**	431 (0.61)	131 (0.70)	1.51	1.07-2.16	0.019
	**Stage**				
***TGFBR2-875G>A***	**Localized (T1-T2 stages)**	**Advanced (T3- T4, N and M+)**			
**AA/AG**	162 (0.39)	166 (0.35)			
**GG**	252 (0.61)	310 (0.65)	1.20	0.91-1.58	0.189
	**Hormone resistance**				
***TGFBR2-875G>A***	**No**	**Yes**			
**AA/AG**	133 (0.40)	55 (0.32)			
**GG**	196 (0.60)	114 (0.68)	1.41	0.95-2.08	0.084

OR, odds ratio; 95% CI, 95% confidence interval; univariate analysis

**Figure 1:**
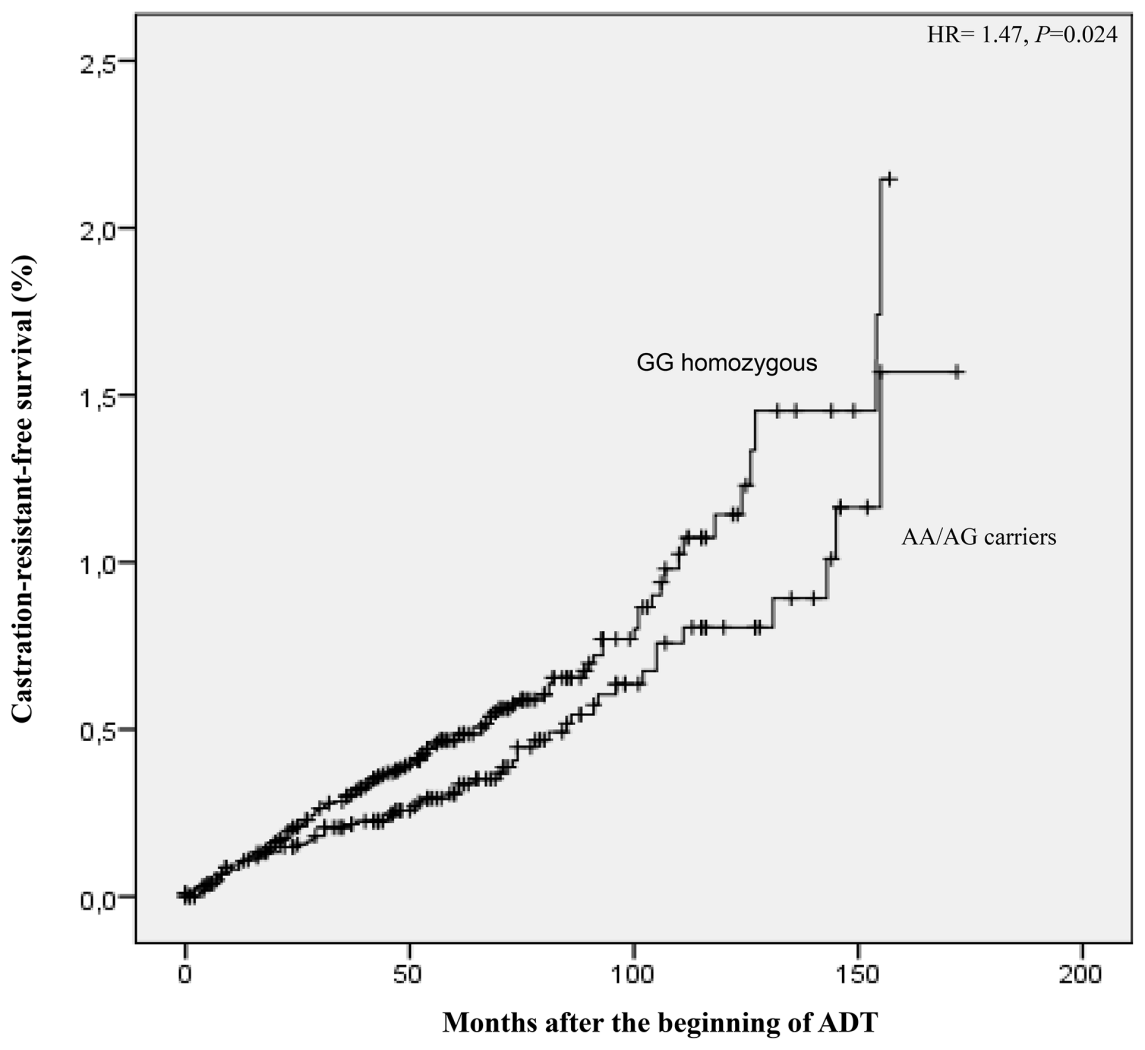
Castration-resistant-free interval (CRFI) according to *TGFBR2-875G>A* genotypes in patients submitted to ADT. Hazard ration using tumor stage (clinically localized *vs* locally advanced *vs* distant metastases, EAU Guidelines), Gleason≥ 8, and PSA levels at diagnosis as covariants.

Concerning the time to CR after the beginning of ADT, we observed that the *TGFBR2-875G>A* polymorphism was associated with CR- free survival. We observed a significantly reduced time-to-CR in *TGFBR2-875GG* homozygous compared with AA/AG genotypes carriers (86.7 *versus* 102.4 months) (Log Rank test, *P*=0.032). Furthermore, multivariate Cox regression model using tumor stage, Gleason≥ 8, and PSA levels at diagnosis as covariants, demonstrated a higher risk of earlier relapse in *TGFBR2-875GG* PC patients following ADT (hazard ratio- HR= 1.47, 95%CI: 1.05-2.05, *P*= 0.024) (Figure 1).

The concordance (c) index was used to compare the predictive ability of different prognostic variables associated with CR development; the predictive value was assessed with Harrell’s concordance indexes, where a c-index of 1 indicates perfect concordance [15]. Tumor stage is a well-known prognostic factor for cancer progression. In our study the predictive value of combined tumor stage (Localized *vs* advanced) for CR development was 0.683 (Model 1). This predictive value increased to 0.736 with the addition of other variables to the predictive model such as PSA ≥ 20 ng.mL^-1^ at diagnosis and Gleason score ≥8 (Model 2). Finally, this predictive ability increased with the addition of genetic information regarding the *TGFBR2-875G>A* polymorphism, with an improvement of the prediction by 6.3% and 1.0% compared with Model 1 and Model 2, respectively (c-index 0.746) (Model 3, Table 3). Furthermore, the predictive value increased to 0.759 considering the covariants tumor Stage (localized *vs* locally advanced *vs* distant metastases, EAU), PSA ≥ 20 ng.mL^-1^ at diagnosis, Gleason ≥8, *TGFBR2-875GG* genotype (Model 4, table 3).

**Table 3:** Predictive models of castration-resistance development after androgen deprivation therapy.

	**HR**	**95% CI**	***P***	***c* index**
**Model 1**				0.683
Combined Tumor Stage (Localized *vs* advanced)	3.89	2.67-5.65	<0.001	
**Model 2**				0.736
Combined Tumor stage (Localized *vs* advanced)	2.61	1.72-3.95	<0.001	
PSA ≥ 20 ng.mL^-1^ at diagnosis	1.47	1.06-2.03	0.020	
Gleason ≥8	1.90	1.40-2.62	<0.001	
**Model 3**				0.746
Combined Tumor Stage (Localized *vs* advanced)	2.70	1.77-4.12	<0.001	
PSA ≥ 20 ng.mL^-1^ at diagnosis	1.53	1.10-2.12	0.012	
Gleason ≥8	1.83	1.32-2.54	<0.001	
*TGFBR2-875GG* genotype	1.39	0.99-1.94	0.058	
**Model 4**				0.759
Tumor Stage (localized *vs* locally advanced *vs* distant metastases, EAU)	2.28	1.78-2.91	<0.001	
PSA ≥ 20 ng.mL^-1^ at diagnosis	1.23	0.87-1.75	0.246	
Gleason ≥8	1.64	1.18-2.29	0.004	
*TGFBR2-875GG* genotype	1.44	1.03-2.02	0.033	

HR, hazard ratio; 95% CI, 95% confidence interval

**Figure 2:**
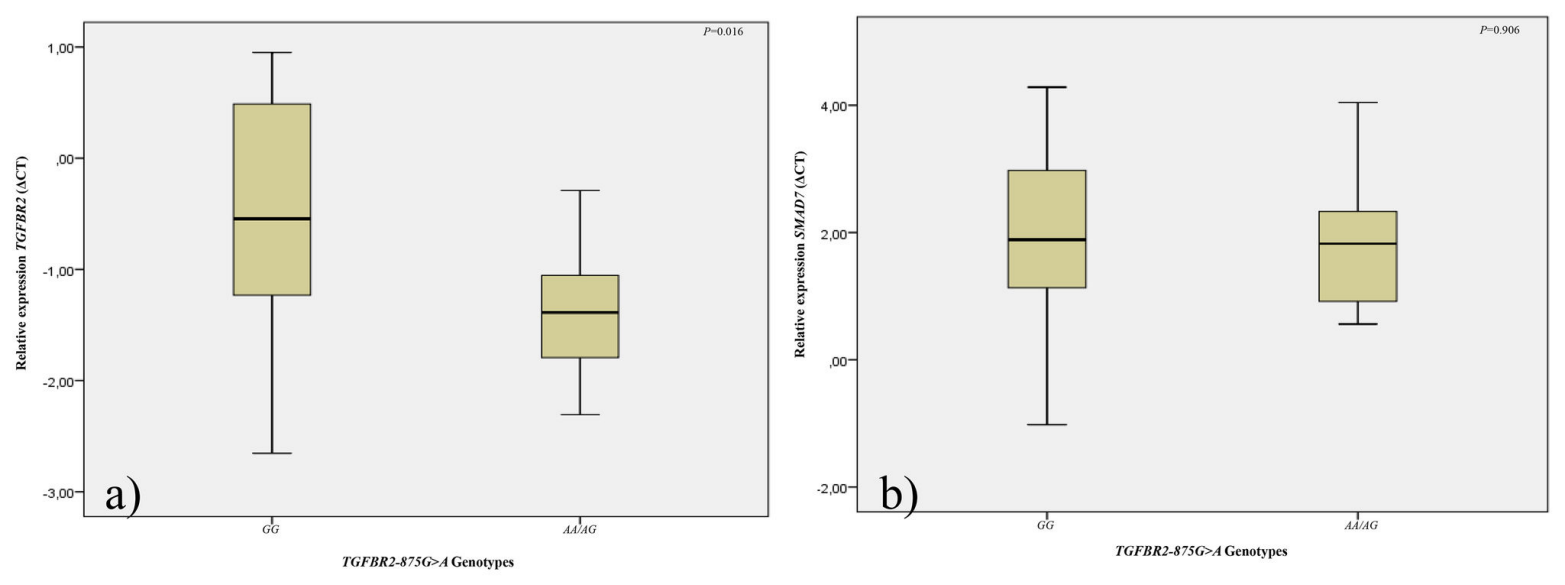
Normalized expression among individuals with *TGFBR2-875GG vs TGFBR2-875*AA/AG genotypes regarding a) *TGFBR2* mRNA and b) *SMAD7* mRNA. Statistical differences in the *TGFBR2* and *SMAD7* mRNA levels were evaluated by the Student’s t-Test.

Regarding the *TGFBR2* mRNA expression according the *TGFBR2-875G>A* polymorphism, we observed differences in mRNA expression in GG homozygous carriers compared with AA/AG genotypes carriers. The AA/AG genotype carriers present a 1.5-fold increase in expression levels of *TGFBR2* mRNA than GG homozygous (2^-ΔΔCT^ = 1.5, *P*=0.016) (Figure 2a). Concerning *SMAD7* mRNA expression, we observed no differences in mRNA expression levels between the *TGFBR2-875GG* homozygous and *TGFBR2-875AA/AG* genotype carriers (*P*=0.906) (Figure 2b).

## Discussion

Worldwide, PC is the most prevalent malignancy in men. Prostate cancer is a complex, multifactorial and heterogeneous disease. A better understanding of the mechanisms involved in this disease may contribute to early diagnosis and to the establishment of new therapeutic targets to increase survival and improve the quality of patients’ life. The PC progression to CR has been associated with the cell insensitivity to anti-proliferative signals and consequent disruption of the balance between cell growth rate and apoptosis. The CRPC is an invariably lethal condition, with chemotherapy being the sole treatment option with only palliative benefits [16,17]. Therefore, it is important to understand the mechanisms involved in CR progression.

In the present study, we describe that the functional *TGFBR2* polymorphism influences the development of CRPC. Our results suggest that small changes in *TGFBR2* expression levels, due to functional SNPs, may contribute to a disequilibrium in the TGFβ1 signaling pathway activation and thus to a higher risk for an earlier development of resistance to ADT.

TGFβ1 signaling pathway mediates growth, mainly by inhibiting cell cycle progression through G1-arrest, suppressing *c-myc*, and stimulating cyclin-dependent kinases inhibitors including p21^WAF1^ and p15^Ink4b^, but also by inducing apoptosis, through the induction of DAP kinase and others [18]. Several studies demonstrated that TGFβ1 signaling components, including TGFβRII, are often lost in human cancer [5]. In PC, the TGFβ1 is frequently up-regulated and the loss of TGFβRI and RII has been detected. Is has been hypothesized, that *TGFBR2* repression is responsible for most of the tumor associated TGFβ1 resistance observed *in vivo* [19]. Studies performed by Rojas and co-workers showed that *TGFBR2* expression levels can regulate the intensity of activation of the Smad and non-Smad signaling pathways [10]. The inactivation of *TGFBR2* gene in mouse fibroblasts has been associated with prostate intraepithelial neoplasia and invasive squamous cell carcinoma of forestomach development, and this alteration can also promote growth and invasion of co-transplanted breast cancer cells [20,21]. Moreover, patients with defective TGFβRII present significantly more colon polyps and preneoplastic lesions than patients with normal TGFβRII [22]. Lu and co-workers, report that the expression of TGFβRII significantly decreased when they observed normal tissue, tissue adjacent to cancer and gastric cancer [23].

Early reports indicate that most PC become resistant to the anti-proliferative effects of TGFβ1 without defined mutations or deletions in members of the Smad signaling pathway [24]. More recently, Rojas and co-workers showed that *TGFBR2* expression levels can affect the ability of TGFβ1 to induce p21 and apoptosis in the V-400 colorectal cancer cell line [10]. Furthermore, cancers that do not demonstrate mutations in any TGFβ1 signaling cascade members, show down-regulated levels of *TGFBR2*, demonstrating the oncogenic potential of TGFβ1 pathway [25].

In the PC3 cell line, which is CR, the AR expression reduces TGFβ1/SMAD transcriptional activity and the growth effects of TGFβ1 (in the absence of DHT), thus preventing growth inhibition and apoptosis. According to Bruckheimer and co-workers, the treatment of LnCaP cells, that overexpress TGFβRII, with TGFβ1 in the presence of DHT, can enhance the cell cycle arrest and apoptosis, through caspase-1 activation and the targeting of BCL-2 [26].

It is evident that TGFβ1 signaling requires a delicate balance of interactions within the cellular and tumoral microenvironment. Deregulation of *TGFBR2* expression levels and of the inhibitory *SMAD7* could influence the normal cellular homeostasis and also influence cancer progression. We previously demonstrated that changes in the tumoral microenvironment can modulate PC progression with impact in response to ADT [13,27].

Seijo and co-workers observed, in normal epithelial cells, that the A-to-G transition at the -875 position enhances the activity of TGFβRII, with higher luciferase activity in the presence of the A allele [12]. Our results also suggest that the *TGFBR2-875G>A* polymorphism could influence the circulating expression levels of *TGFBR2* in PC patients. We observed that T*GFBR2-875>AG/AA* patients carriers present a higher expression levels of *TGFBR2* mRNA than GG homozygous (2^-ΔΔCT^ = 1.5, *P*=0.016) and that up-regulation may be responsible for the TGFβ1 signaling pathway up-regulation, inducing the cell cycle arrest and apoptosis, and consequently causing these patients to present a higher progression free-interval after the beginning of ADT (AA/AG: 102.4 *vs* GG: 86.7 months).

In fact we also observed that *TGFBR2-875GG* homozygous, which present lower circulating levels of *TGFBR2* mRNA, present a higher risk for the development of tumors with higher Gleason score (≥8) (*P*=0.019). In GG homozygous carriers, the lower expression levels of *TGFBR2* may be associated with cellular disruptions in the TGFβ1 signaling pathway and induces the acquisition of aggressive cancer phenotypes.

*TGFBR2-875>G/A* has been the subject of investigation in several case–control studies involving different types of cancers, with controversial results [28–32].

Several authors, have associated the *TGFBR2-875G>A* polymorphism with a decreased risk for gastric cancer, esophageal squamous cell carcinoma, breast cancer, and lymph node metastasis, especially in the Asian population [28–33].

However, similarly to other studies in European populations, no association were observed between the *TGFBR2-875/A* polymorphism and PC risk. In addition, it appears that this polymorphism may have no influence on the onset of PC but instead it can modulate its progression to an aggressive phenotype [34].

In our control population the frequency of the *TGFBR2-875G>A* polymorphism tended to be similar to those observed in other Caucasian healthy populations like in Finland and Poland. However, the frequencies are also similar to those observed in studies performed in Republic of Korea and North of China [30,33,34]. Additionally, we observed that the frequency of the *TGFBR2-875GG* genotype was lower than the one of the control group used in the study performed by Seijo and co-workers in an American population and higher than the reported results in the control group that included Cantonese healthy subjects from population in southern China [12,32].

Survival of the PC patients and CR development are related to several variables that include the extent of the initial tumor, PSA levels, patients’ age, which are useful tools for therapeutic decisions [14]. However, due to some predictive limitations, they do not exactly reflect the biologic behavior of PC, and many men can die from aggressive disease. Additional results showed that the definition of profiles that contains information regarding tumor characteristics associated with genetic information have a higher capacity to predict the risk for CR development (c-index from 0.683 to 0.746). Therefore, we hypothesize that the definition of a predictive profile that contains the *TGFBR2-875G>A* polymorphism information could be useful as molecular marker for predicting the clinical response to ADT in patients with PC. However, future studies should replicate this predictive model in another PC study population submitted to ADT.

In conclusion, the present study suggests that the functional polymorphism *TGFBR2-875G>A* does not modify the predisposition to PC in our population. However, this SNP has the capacity to change the *TGFBR2* mRNA expression levels in PC patients submitted to ADT and influence the CRFI, with a lower time interval to CR in *TGFBR2-875GG* homozygous compared with *TGFBR2-875AG/AA* patients carriers. In the future, the identification of these genetic profiles may help to define new effective prognostic models for the follow up of patients submitted to ADT and identifying higher risk groups for an individualized chemoprevention strategy and target therapies.
