# Correction: Improvement of a Predictive Model of Castration-Resistant Prostate Cancer: Functional Genetic Variants in TGFβ1 Signaling Pathway Modulation

**DOI:** 10.1371/annotation/47bdb3be-4110-48de-a29d-e4734e64f6a0

**Published:** 2013-08-21

**Authors:** Ana L. Teixeira, Mónica Gomes, Augusto Nogueira, Andreia S. Azevedo, Joana Assis, Francisca Dias, Juliana I. Santos, Francisco Lobo, António Morais, Joaquina Maurício, Rui Medeiros

There was an error in the Funding statement. The correct statement is available below:

The authors would like to thank the Liga Portuguesa Contra o Cancro-Centro Regional do Norte (Portuguese League Against Cancer) and Fundação para a Ciência e Tecnologia (FCT), this project was partially sponsored by an unrestricted educational grant for basic research in molecular oncology from FCT (PTDC/SAU-FCF/71552/ 2006). This project was partially funded by FCT and by Operational Programme "Factores de Competitividade" (COMPETE) (PTDC/SAU-FC/71552/2006 and FCOMP-01-0124-FEDER-011113). ALT is a Doctoral degree grant holder from FCT (SFRH/BD/47381/2008). The funders had no role in study design, data collection and analysis, decision to publish, or preparation of the manuscript. 

